# Mullerian adenosarcoma accidentally detected and coexisting with cervical carcinoma *in situ*: a rare case report

**DOI:** 10.3389/fonc.2024.1482768

**Published:** 2024-10-10

**Authors:** Xuemei Qing, Min Xie, Hongying Guo, Liying Zhang, Jiatian Ye, Yong Zhang, Ying Ma

**Affiliations:** ^1^ Department of Obstetrics and Gynecology, People’s Hospital of Qingbaijiang District, Chengdu, Sichuan, China; ^2^ Department of Obstetrics and Gynecology, Southwest Medical University, Luzhou, Sichuan, China; ^3^ Department of Obstetrics and Gynecology, West China Second University Hospital, Sichuan University, Chengdu, Sichuan, China; ^4^ Department of Obstetrics and Gynecology, Mianyang Central Hospital, Mianyang, Sichuan, China; ^5^ Department of Obstetrics and Gynecology, Chengdu Medical College, Chengdu, Sichuan, China

**Keywords:** Mullerian adenosarcoma, cervical carcinoma in situ, multiple primary malignant neoplasms, rare, case report

## Abstract

Mullerian adenosarcoma is rare, usually found in the uterine corpus and rarely in the cervix. Adenosarcoma that grows diffusely in the uterine cavity and the cervical canal is even rarer without symptoms. Herein, we report a rare case of multiple primary malignant neoplasms of Mullerian adenosarcoma accidentally detected and coexisting with cervical carcinoma *in situ*. Fortunately, the tumor was in the early stage and the Mullerian adenosarcoma was treated together with the cervical carcinoma *in situ* by hysterectomy + bilateral adnexectomy. Histopathology and immunohistochemistry results confirmed this diagnosis, further confirmed by a pathology consultation at the University Hospital. The patient recovered well from the surgical treatment and was discharged with regular follow-up. The patient did not undergo pelvis–abdomen CT and diagnostic curettage preoperatively, and no malignancy was detected by cryo-pathology intraoperatively, which may be related to the rarity of the disease and the relative lack of awareness and experience of our clinicians and pathologists for this tumor. We hope that this rare case can provide some lessons for gynecologists and pathologists.

## Introduction

Mullerian adenosarcoma (MA), also known as uterine adenosarcoma, is a rare mixed tumor consisting of benign epithelial and malignant mesenchymal components, which occurs predominantly in the uterus (approximately 81%) and rarely in the cervix (approximately 2%) (1). It varies in clinical symptoms and may be asymptomatic. Cervical intraepithelial neoplasia (CIN) III with glandular involvement, equivalent to cervical carcinoma *in situ* (CCIS), often caused by persistent high-risk human papillomavirus (HPV) infection, manifests primarily as vaginal bleeding following coitus but some may also be asymptomatic. Multiple primary malignant neoplasms (MPMN) are even rarer, but with the increase in human life expectancy and the advancement of medical technology, the rate of early cancer detection has increased and the reports about MPMN also increased (2, 3). Now, we report a rare case of asymptomatic CCIS coexisting with MA as an MPMN.

## Case presentation

The patient, a 56-year-old woman, G_0_P_0_, menopause for 8 years, was admitted to our hospital for “Abnormal cervical cancer screening results detected for more than 3 months”. Past medical history and family history were not specific; she had a middle school education, had freelance work, and drank in moderation for 40+ years. She denied postmenopausal vaginal bleeding and discharge, without abdominal pain, or bloating. More than 3 months ago, she had a health checkup with positive HPV-58 and an abnormal Thin-prep cytologic test about atypical squamous cells. Eleven days before admission, a cervical biopsy was carried out, and the results showed that “Cervix 6 point” and “Cervix 12 point” had foci of high-grade squamous intraepithelial neoplasia (CIN III) with glandular involvement, considered CCIS, and surgery was recommended.

**Figure 1:**
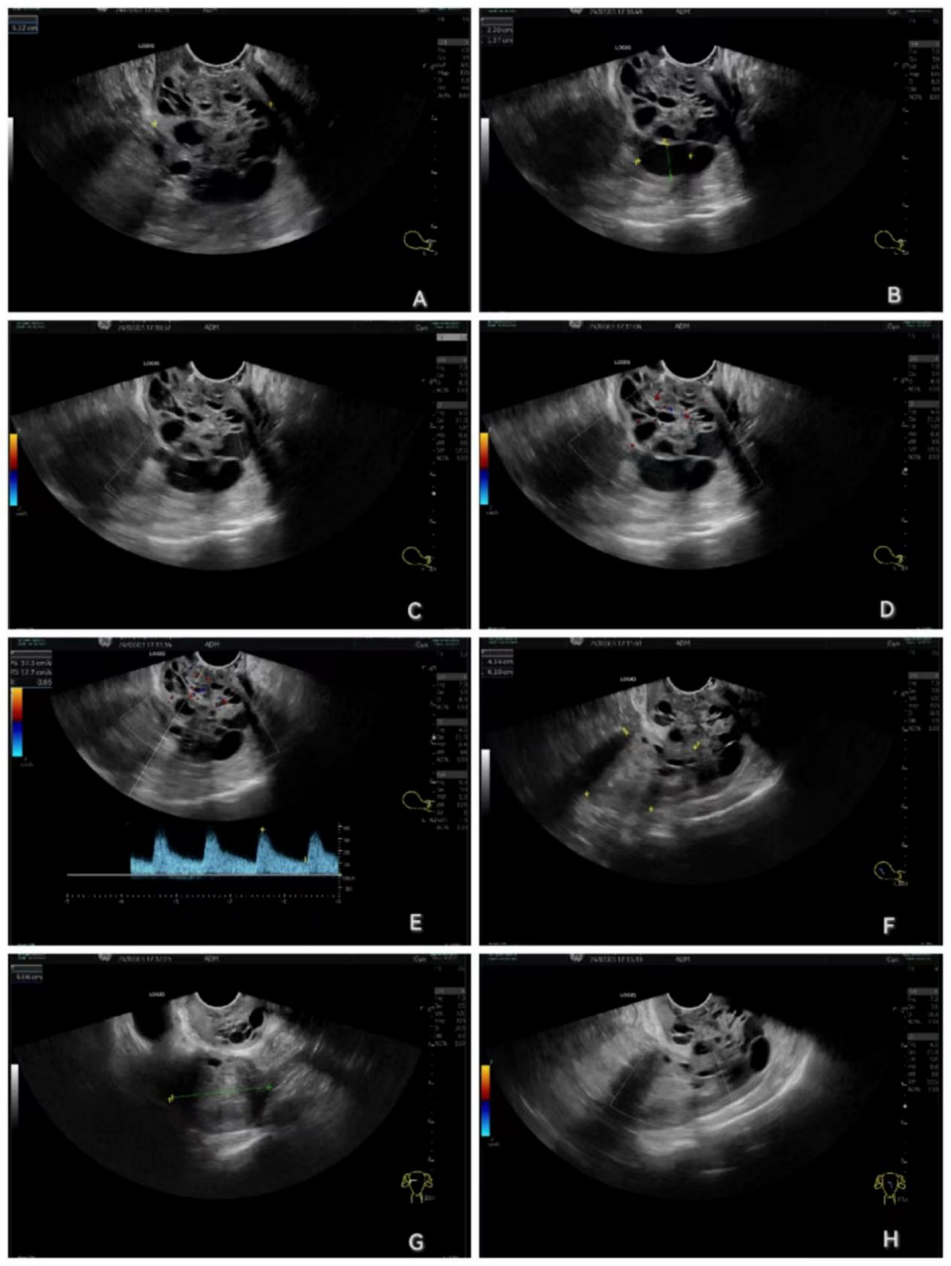
Transvaginal ultrasound images. **(A)** The cervix was thickened with an anteroposterior diameter of 4.3 cm and multiple cystic echoes. **(B)** The largest cystic echo of the cervix was approximately 2.2 cm x 1.4 cm. **(C)** Maximum cystic echoes of the cervix show no blood flow signal. **(D)** Punctate blood signals throughout the cervix. **(E)** RI: 0.66. **(F)** The anteroposterior diameter of the uterus was 4.3 cm with a longitudinal diameter of 6.1 cm. **(G)** The transverse diameter of the uterus was 5.1 cm. **(H)** The endometrium is poorly visualized without a blood flow signal but with the arterioles in the surrounding area.

Physical examination showed the following: temperature, 36.3°C; pulse rate, 68 times/min; respiratory rate, 20 times/min; blood pressure, 100/62 mmHg; weight, 62.5 kg; height, 151 cm. There were no positive signs in the general examination. However, gynecological examination showed cervical atrophy with post-biopsy changes, visible heterogeneous vessels, and uterine atrophy slightly, with no masses and pressure pain detected in the bilateral adnexal area. Pathologic results of cervical biopsy were as follows: High-grade squamous intraepithelial neoplasia (CIN III) with glandular involvement was seen in both “Cervix 6 point” and “Cervix 12 point”. Preliminary diagnosis showed CIN III with glandular involvement and high-risk HPV infection. She underwent a series of preoperative ancillary examinations, in which a transvaginal ultrasound (**Figures 1A-H**) showed that the cervix was thickened with an anteroposterior diameter of 4.3 cm and multiple cystic echoes, the largest one being approximately 2.2 cm x 1.4 cm without blood flow signal, and punctate blood signals throughout the cervix was detected with an RI of 0.66. The anteroposterior diameter of the uterus was 4.3 cm with a longitudinal diameter of 6.1 cm and a transverse diameter of 5.1 cm. The endometrium is poorly visualized without a blood flow signal but with the arterioles in the surrounding area, no mass in the bilateral adnexal area, and no free echogenicity in the pelvis. Other results showed no abnormalities, including chest computed tomography (CT), which was required before elective laparoscopic surgery at our hospital.

**Figure 2:**
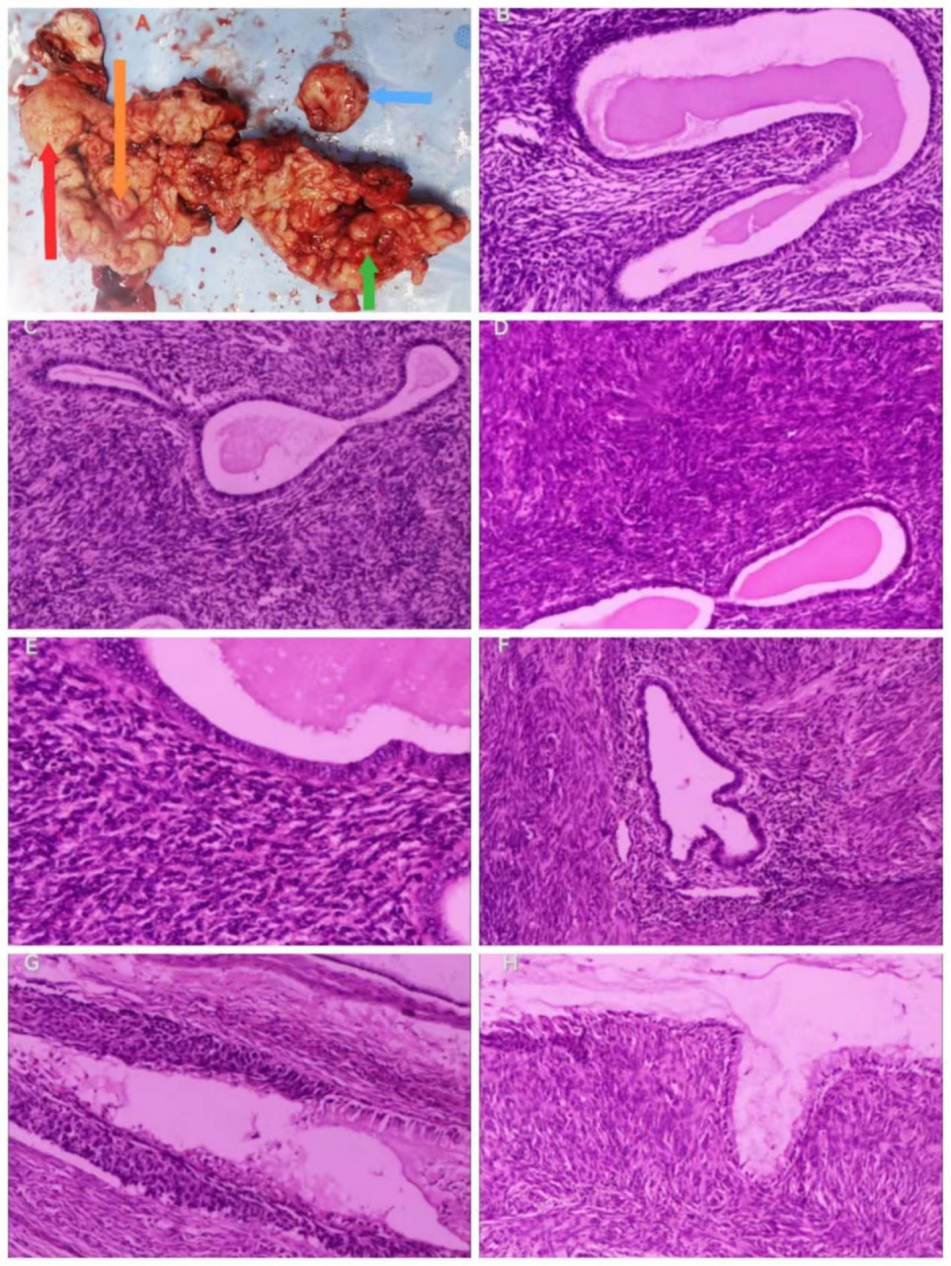
Gross view of the specimen and histopathologic results (hematoxylin–eosin staining). Gross view of the specimen. **(A)** The arrows point to red: adenomyosis, orange: uterine adenosarcoma, green: cervical adenosarcoma, and blue: the largest cystic echogenic mass of the cervix). Histopathologic results. **(B)** Irregular endothelial glands, 100x. **(C)** (100x), **(D)** (100x), and **(E)** (200x): Dense mesenchyme surrounding endothelial glands. **(F)** Adenomyosis, 100x. **(G)** CIN III with glandular involvement, 100x. **(H)** Dense mesenchyme around the cervical glands, 100x.

Then, because of obvious atrophy of the cervix after years of menopause, because cervical conization proved to be difficult, and because there was no desire to preserve her uterus, a trans-umbilical single-port laparoscopic hysterectomy + bilateral adnexectomy was performed. Intraoperative observation: the uterus anterior was slightly larger than normal, spherical, with no tumor on the corpus surface, but one mass approximately 3 cm x 4 cm in size was seen at the junction of the lower part of the posterior wall and the cervix, with a smooth surface, and the bilateral adnexa had no abnormality in appearance. The gross view of the isolated uterus specimen profile (**Figure 2A**): No neoplasm was seen on the surface of the external cervix, the cervix was significantly thickened with a diameter of approximately 5 cm, with multiple cystic cavities containing mucus, located mainly in the posterior wall, and the muscular wall of the uterus was not uniform, with many small gray-white papillomatous nodules, diffused in the uterine cavity. The results of cryo-pathology showed the following: chronic cervical and endocervical inflammation with retention cysts, small foci of CIN III with glandular involvement, and multiple grayish-white polypoid protrusions in the uterine cavity, which tended to be adenomyoma-like polyps. Thus, with no malignant lesions seen, the operation was over.

**Figure 3:**
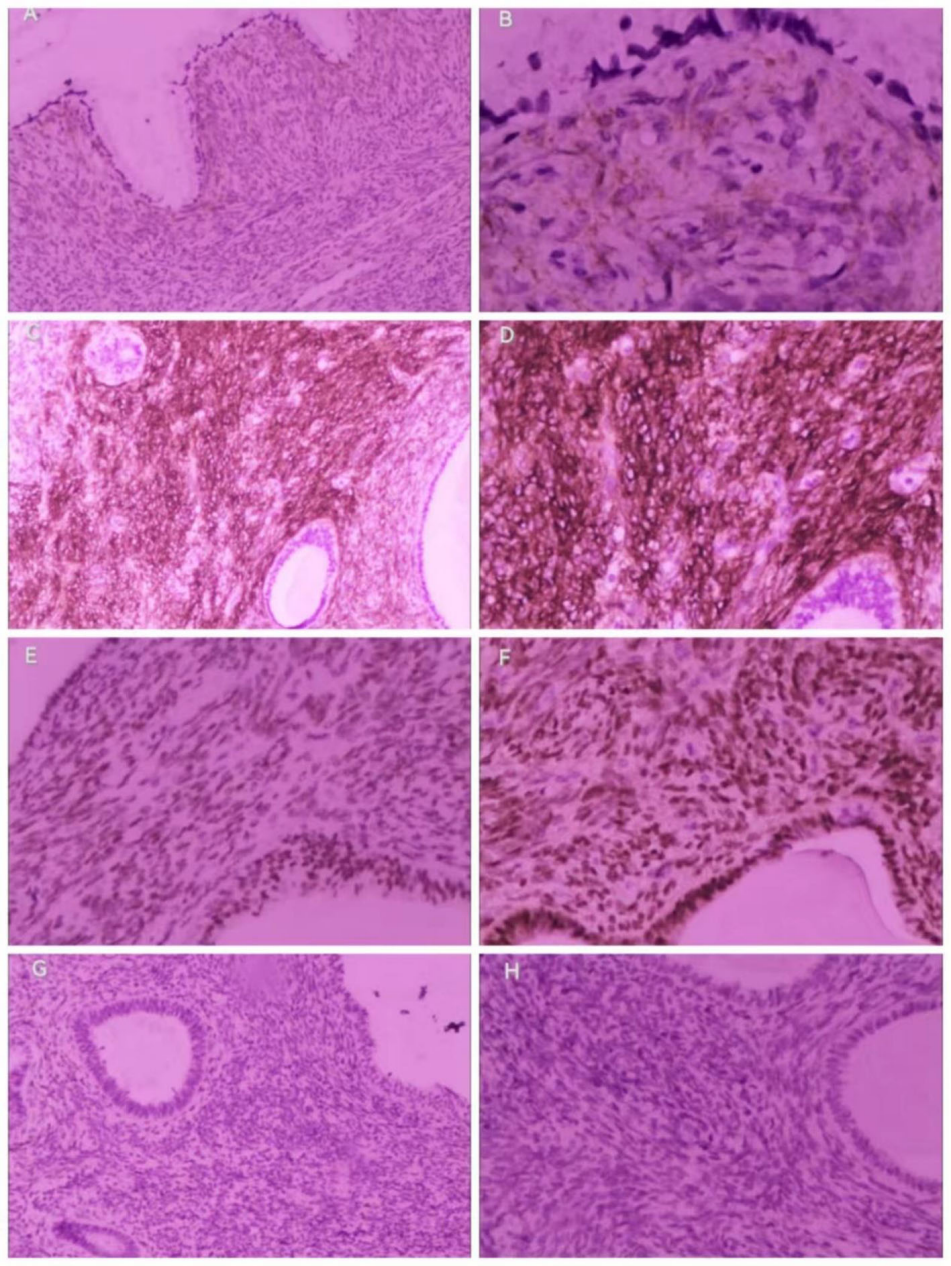
IHC of the adenosarcoma. Dense mesenchyme surrounding cervical glands: CD10 (+), **(A)**: 100x; **(B)** (400x). Dense mesenchyme surrounding endometrial glands: CD10 (+), **(C):** 100x; **(D)** (:200x); **(E)** ER (+), 200x;**(F)** PR (+), 200x; **(G)** Desmin (−), 100x; **(H)** SMA (−), 100x.

The pathological results showed the following: “Uterus” (**Figures 2B-H**) (1): Endometrial glandular dilatation, dense peri-endometrial glandular–mesenchymal stroma with polypoid changes, considered a mixed epithelial–mesenchymal tumor of the uterine corpus, low-grade uterine adenosarcoma, but extensive sampling and immunohistochemistry were required (2); chronic cervical and endocervical inflammation with trapping cyst formation, with small foci of epithelium showing CIN III involving glands, another small focal peri-epithelial mesenchymal hyperplasia of the cervical canal adenohypophysis was seen enthusiastically, and it was considered a mixed epithelial–mesenchymal tumor of the uterine corpus involving the cervix. “Bilateral adnexa”: bilateral ovarian leucoplast and both fallopian tubes chronic inflammation, with interstitial vascularization and congestion. The results of immunohistochemistry (IHC) (**Figures 3A-H**) showed the following: Wax Block No. 17: endometrial gland peritubular dense mesenchymal stroma showed ER (+), PR (+), CD10 (+/−), SMA (−), Desmin (−), P53 (−), and Ki-67 (+; hot spot area of approximately 5%); Wax Block Nos. 10, 11, 12, 14, and 20: peritubular dense mesenchymal stroma were all CD10 (+). The combination of hematoxylin–eosin staining morphology and IHC findings supported a uterine mixed epithelial–mesenchymal tumor predisposing to low-grade uterine adenosarcoma involving the muscle wall and cervix. Differential diagnoses include (1) adenomyoma-like polyps with adenomyosis (2), mixed endometrial–cervical polyps with adenomyosis, and (3) low-grade endometrial mesenchymal sarcoma with gland differentiation. A pathology consultation at a higher hospital was recommended. Then, the consultation results of West China Second Hospital of Sichuan University (pathology no. JCS202403489) showed the following: <Uterine cavity protruding> uterine adenosarcoma, sarcoma component is low-grade endometrial mesenchymal sarcoma, the tumor grows downward and involves the cervix, and adenomyosis foci can be found in the attached muscle wall; <cervix> CIN II and glandular involvement were also seen. IHC: sarcoma component: Ck-P (−), EMA (−), Vim (+), Des (+), Caldesmon (−), CD10 (++), ER (++), PR (−), and Ki-67 positivity <1%.

**Figure 4:**
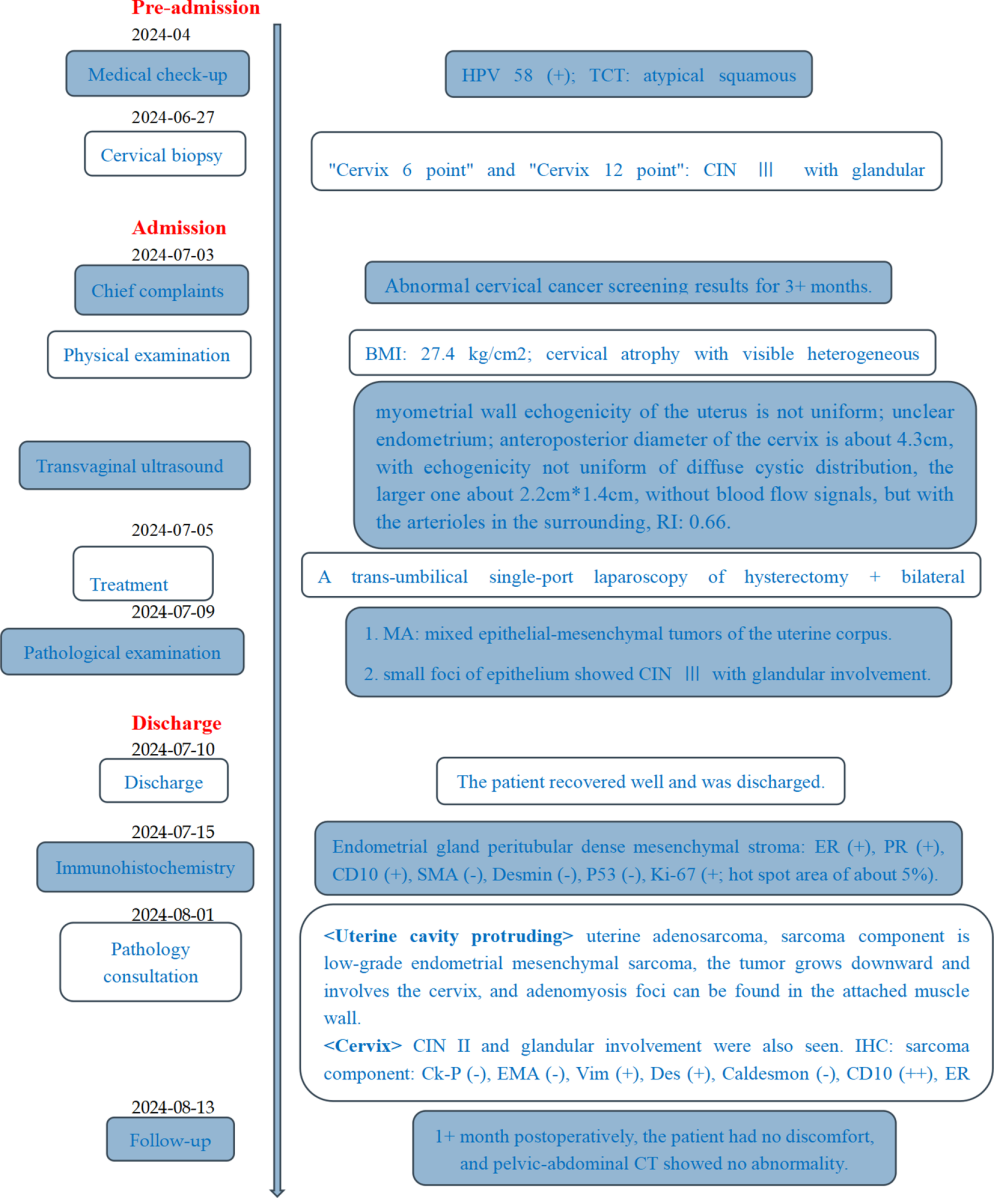
Timeline. HPV, human papillomavirus; TCT, thin-prep cytologic test; CIN, cervical intraepithelial neoplasia; BMI, body mass index; RI, resistance index; MA, Mullerian adenosarcoma.

On the fifth postoperative day, she recovered well and was discharged. About 1 month later, she came for a follow-up with no abnormality of the pelvic–abdominal CT (**Figure 4**). She will continue to be followed up regularly.

## Discussion

MPMNs refer to the simultaneous or sequential appearance of two or more histologically unrelated primary malignant lesions in the same body, which may originate from the same organ, paired organs, different parts of the same system, or organs of different systems, accounting for 0.52%–11.7% of all malignant tumors. MPMN originating in the female reproductive system is relatively rare, accounting for 1%–2% of gynecologic malignant tumors. The pathogenesis is still unclear and may be related to genetic factors, host susceptibility, immune deficiencies, hormonal factors, unhealthy lifestyles, and oncogenic side effects from previous cancer treatments and even environmental exposure (2). It can be divided into synchronous MPMN with occurrence time intervals <6 months and heterochrony MPMN with intervals >6 months, accounting for approximately 90% of cases (4). This patient was diagnosed with CCIS and MA within 1 month of each other, with different primary sites and pathologic types, consistent with synchronous MPMN, which is a rare type. High-risk HPV infection led to her CCIS, and early diagnosis was achieved by combined cervical lesion screening; thus, the treatment was performed promptly. However, one study has shown that HPV is not associated with MA (5).

MA is rare and usually lacks specificity, commonly in postmenopausal women (mean age of 58 years), but it can develop before menopause or even during adolescence (30%). The most common symptom is abnormal vaginal bleeding, and some may present with increased vaginal discharge or pelvic pain. Typically, the tumor grows in the uterine cavity of an exophytic polyp form, which may be located in the lower part of the uterine cavity, and rarely in the cervical or extra-uterine area. Gynecologic color Doppler ultrasound is the most used imaging modality, with a limited differential diagnostic value. In contrast, magnetic resonance imaging (MRI) can provide more details: the tumor is mass-shaped, with clear and sharp borders, intra-tumor heterogeneity, multiple microscopic vesicle shadows, rich blood supply, and even shadows of flow vessels, and the degree of enhancement of the tumor increases with increasing tumor diameter, with higher diffusion-weighted imaging signals and lower apparent diffusion coefficient values. In addition, diffusion-weighted imaging is useful for tumor localization and characterization. Although diagnostic curettage or endometrial biopsy may help diagnose some MA, the sensitivity is poor. It is difficult to differentiate the benign or malignant nature of the tumor preoperatively, by either ultrasound, CT, or MRI, and the diagnosis relies on pathophysiologic examination.

For MA incidentally found after hysterectomy, chest/abdominal/pelvic CT or abdominal/pelvic MRI and chest CT should be performed for the presence of metastatic lesions (6). This patient was diagnosed with CIN III with glandular involvement, and because of obvious atrophy of the cervix, because cervical conization proved to be difficult, and because there was no desire to preserve her uterus, hysterectomy + bilateral adnexectomy was performed (7), with cryo-pathology intraoperatively, but without preoperative pelvic/abdominal CT or MRI. Early-stage cervical cancer is usually asymptomatic, emphasizing the importance of screening diagnosis based on histology. Diagnostic suspicion in initial cases is based on a suspicious or positive Pap test report, followed by a colposcopy, which allows a biopsy to be performed. If insufficient to determine invasiveness, a cervical conization may be required. The tumor marker squamous cell carcinoma antigen has not been evaluated in all international guidelines except the Italian AIOM guidelines. The latest evidence supports the surgical treatment of patients with early-stage cervical cancer in stages not more than IA1, where both cervical conization and extra-fascial hysterectomy can be performed without lymphadenectomy (8). A systematic review of 18 studies demonstrated the importance of a multidisciplinary approach to cervical cancer patients, tailoring the best treatment plan for them, informing them about possible postoperative vaginal changes and menopausal symptoms, and providing them with the best possible psychological support, as well as encouraging a balanced diet and physical activity (9).

In addition, transvaginal ultrasound showed that her endometrium was poorly visualized, which was mistaken for possible endometrial adhesions due to low estrogen levels, and the cervix was enlarged with an anteroposterior diameter of approximately 4.3 cm with multiple cystic echoes, which was mistaken for cervical natriuretic cysts; thus, diagnostic curettage or endocervical curettage was not performed. However, intraoperative dissection with the isolated uterus revealed the presence of diffuse exophytic papillae in the uterine cavity and cervical canal, which aroused the interest of all operators, whether it is an endometrial malignancy with infiltration of the cervix or a cervical invasive carcinoma spreading to the endometrium. However, the pathology report negated both of the above hypotheses. It revealed a rare uterine adenosarcoma (both uterus and cervix involved) with no infiltrative component of CCIS, as determined by our gynecologic pathologist through extensive sampling, careful microscopic examination, and a combination of IHC, literature review, and departmental discussion. The patient’s uterine adenosarcoma was stage I, and hysterectomy + bilateral adnexectomy was the standard procedure, without additional surgery, radiotherapy, chemotherapy, or tumor endocrine therapy (i.e., tumor hormone therapy, which refers to the method of controlling and treating tumors by altering the balance of the body’s endocrine environment, mainly including anti-estrogen and anti-progesterone treatment).

MA has a very low incidence, a rare biphasic tumor with unclear pathogenesis and etiology, which may be associated with several high-risk factors, such as overload or imbalance of estrogen, and use of oral contraceptives or tamoxifen for a long period; high body mass index; and history of pelvic radiation therapy (1). The patient has none of the above high-risk factors, except for a high body mass index of 27.4 kg/cm^2^, close to obesity, which causes various metabolic diseases and is also an important risk factor for endometrial cancer. It is worthwhile for clinicians to pay enough attention, especially perimenopausal and postmenopausal women. In addition, the patient’s ultrasound suggested that the myometrial wall echogenicity is not uniform, which was also confirmed intraoperatively. Whether the tumor infiltrated the myometrial wall microscopically or a manifestation of adenomyosis posed a difficult problem for our pathologists to distinguish it from adenomyoma-like polyp with adenomyosis, mixed endometrial–cervical polyp with adenomyosis, or low-grade endometrial mesenchymal sarcoma with gland differentiation. Furthermore, the report from the gynecologic pathologist at the university hospital confirmed that “adenomyosis foci were detected in the attached muscle wall”. Thus, it is reasonable to wonder whether the presence of adenomyosis is one of the risk factors for MA, or whether MA is a malignant transformation of adenomyosis, which is based on evidence (10, 11). Additionally, a recent report shows that endometriosis may be a factor in the development of MA (12).

The diagnosis of MA relies on histopathology, with the following features: a mixed tumor composed of benign neoplastic glandular and sarcomatous mesenchymal components that are lobulated and may be seen as fissured or dilated glands lined by benign endometrial epithelium, with cuff-like neoplastic mesenchymal cells surrounding the glands, which are abundant and show varying degrees of anisotropy, and the nuclear schizophrenic phenomenon is generally rare or absent. The malignant mesenchymal component is usually low grade and consists of spindle-shaped and/or rounded cells, the former often arranged in whorls, the latter loosely arranged, with the mesenchymal cells clustered around the gland and sparsely cuffed away from the gland, forming the so-called germinal layer. The sarcoma component is homogeneous, showing either endometrial mesenchymal or smooth muscle differentiation, when the overall prognosis of the tumor is better than that of other sarcomas of the uterus. When the growth of the mesenchymal sarcoma component significantly exceeds the gland component, meaning that the pure sarcoma component accounts for more than 25% of the tumor volume and that the cellular abnormality increases, presenting high-grade sarcoma manifestations or the appearance of rhabdomyosarcoma and other heterogeneous differentiation, it is called adenosarcoma with sarcomatous overgrowth (SO), with high invasiveness and poor prognosis (13). IHC is not mandatory, but low-grade adenosarcomas are usually positive for CD10 and hormone receptors, and their mesenchymal components resemble endometrial mesenchymal sarcomas immunohistochemically. p53 shows aberrant expression in the overgrown sarcoma component, and its mesenchymal components resemble high-grade uterine sarcomas with a high Ki-67 index. Desmin, myogenin, and MyoD1 help identify rhabdomyosarcoma components (14). In this patient, immunohistochemistry, as well as consultation with the pathologist at the university hospital, showed that the adenosarcoma originated in the uterine cavity, the adenosarcoma in the cervical canal was due to a downward invasion of the uterine lesion, the lesion was low grade, and no sarcoma overgrowth was seen as demonstrated in the pathology report (p53-negative, low positivity for Ki-67, etc.).

With the development of molecular pathology, several genetic tests have been applied to MA. Their molecular characteristics lack features such as specific gene mutations or chromosomal variants; some are seen with *8q13* amplification and *MYBL1* copy number increase associated with SO, and few are seen with *NCOA2/3* gene fusion. It has been shown that the genomic and clinicopathologic features of high-grade MA include generalized *TP53* pathway alteration and aggressive behavior (15). A recent study showed that in 29 MA cases among 40 commonly mutated genes detected by whole-genome sequencing and verified by next-generation sequencing, *KMT2C* and *BCOR* were common with and without SO, whereas *MAGEC1* and *KDM6B* were strongly associated with SO, and that the frequent gene mutation rate of approximately 33% of MA with SO was higher than that without SO (11%) (16).

Surgery is the mainstay of treatment, supplemented by endocrine therapy, chemotherapy, and radiotherapy. Systematic pelvic and para-aortic lymph node dissection is not routinely performed, but exploration is needed, and enlarged or suspicious lymph nodes should be removed. If the lesion is confined to the uterus (1): hysterectomy + bilateral adnexectomy; (2) inoperable: external pelvic radiation ± brachytherapy and/or systemic treatment. If extrauterine: (1) hysterectomy + bilateral adnexectomy + resection of metastatic foci, including metastatic lymph nodes; (2) inoperable: extra-pelvic irradiation ± brachytherapy and/or systemic treatment. The complete removal of the uterine tumor is emphasized, and tumor fractionation in the peritoneal cavity is contraindicated. The supplemented therapy depends on the disease stage after surgery: (1) Adenosarcoma without sarcoma overgrowth: stage I can be observed or estrogen blockade; stages II–IV_A_ with anti-estrogen therapy and/or radiotherapy; stage IV_B_ with anti-estrogen therapy and/or palliative radiotherapy; (2) adenosarcoma with sarcoma overgrowth: stage I can be observed, and stage II–IV needs chemotherapy (6). Clinically, corrections based on clinicopathologic prognostic factors are often required, and a review by the gynecologic pathologist is strongly recommended. Relevant risk factors include hysterectomy, completeness of tumor specimen, tumor size, histologic type, number of nuclear schizonts, presence of vascular infiltration, myometrial invasion, and histologic grade. In addition, the location and number of extrauterine metastases should be recorded in detail, and the number and location of lymph nodes involved should be clarified.

MA is of low malignant potential and has a relatively good prognosis, but still, 25% of patients die. Poor prognostic factors include sarcoma overgrowth, deep mesenchymal infiltration, high-grade component, high heterogeneous component, and lympho-vascular infiltration, of which the first two are associated with the worst prognosis (14). Local recurrence and distant metastases occur in approximately 5%. Regular follow-up is important, including general and gynecological examinations, imaging tests, and health education.

## Conclusions

MA is rare, and even rarer coexisting with CCIS as an MPMN. The patient did not undergo pelvis–abdomen CT and diagnostic curettage preoperatively, and the cryo-pathology failed to detect the MA, which may be related to the rarity of the disease and the lack of experience of our clinicians and pathologists. Fortunately, it is stage I and the hysterectomy + bilateral adnexectomy surgical procedure was suitable, without additional surgery or other supplemented therapy. Raising awareness for MA and MPMN to reduce misdiagnosis and underdiagnosis is profoundly important for the diagnosis and treatment.

## Data Availability

The datasets presented in this study can be found in online repositories. The names of the repository/repositories and accession number(s) can be found in the article/supplementary material.
